# Sarcomatoid carcinoma of the common bile duct: A case report and review of the literature

**DOI:** 10.1007/s13691-026-00852-8

**Published:** 2026-03-10

**Authors:** Sedat Alp Pinar, Mari-Claire McGuigan, Fraser Duthie, Abinaya Ezhil, David Holroyd, Nigel B. Jamieson

**Affiliations:** 1 Academic Unit of Surgery and Lister Department of Surgery, Glasgow Royal Infirmary, University of Glasgow, Glasgow, G31 2ER UK; 2 Wolfson Wohl Cancer Research Institute, School of Cancer Sciences, University of Glasgow, Glasgow, G12 8LE UK; 3 Department of Pathology, Laboratory Medicine Building, Queen Elizabeth University Hospital, University of Glasgow, Glasgow, G51 4TF UK; 4 Department of Radiology, Glasgow Royal Infirmary, Glasgow, G31, 2ER UK

**Keywords:** Hepatopancreatobiliary surgery, Bile duct malignancy, Common bile duct, Sarcomatoid carcinoma, Rare malignancy, Immunohistochemistry, Case report

## Abstract

Sarcomatoid carcinoma of the common bile duct is an exceptionally rare malignancy, with only eight cases previously reported in the English literature. We present the case of a 72-year-old Caucasian male who presented with obstructive jaundice and abnormal liver function tests, but without elevated tumour markers CA19-9 and CEA. Imaging with CT and MRCP revealed a polypoid mass in the common bile duct, and two ERCP attempts were unsuccessful. The patient underwent a pancreaticoduodenectomy without adjuvant chemotherapy and has remained recurrence-free for 16 months. Histopathology revealed sarcomatoid spindle cells negative for the expression of sarcomatous markers but positive for epithelial markers CAM5.2 and AE1/3, as well as MNF focally, along with chronic cholecystitis. Sarcomatoid carcinoma is a rare and challenging diagnosis that requires tissue confirmation and immunohistochemical staining for accurate identification. Despite its rarity, it is important to consider sarcomatoid carcinoma in the differential diagnosis of polypoid masses of the common bile duct, especially in the absence of elevated tumour markers. There is inconclusive evidence regarding the clinical outcomes for sarcomatoid carcinoma of the common bile duct and the effectiveness of various treatment strategies. Current literature indicates the widespread use of pancreaticoduodenectomy; while the role of chemotherapy remains unclear.

**Supplementary Information:**

The online version contains supplementary material available at 10.1007/s13691-026-00852-8.

## Background

Sarcomatoid carcinoma is an exceptionally rare and aggressive tumour affecting the hepato-pancreato-biliary system, characterized by the presence of giant cells/spindle cells with sarcomatous morphology with an epithelial origin [1, 2].

Sarcomatoid carcinoma has been reported in the liver [3], gallbladder [4], pancreas [5], and ampulla of Vater [6]. However, occurrences in the common bile duct are exceptionally rare. This rarity poses a significant challenge in studying these tumours and establishing a standardized treatment protocol.

The origin of sarcomatoid carcinoma is not well understood, though three main theories have been proposed [6]. According to Kench and Frommer (1997), sarcomatoid carcinoma may develop through a metaplastic transformation of carcinomatous cells into sarcomatous cells [6]. Another possibility is that it arises from a single multipotent reserve cell that has the ability to differentiate in various ways [6]. A third theory suggests that sarcomatoid carcinoma results from the simultaneous malignant growth of both epithelial and stromal cells [6].

While these tumours are generally regarded as highly aggressive with a tendency for high recurrence and poor survival, the limited literature provides mixed survival outcomes, underscoring the need for more comprehensive research and case studies [7]. We believe the aggressive nature of these tumours may be associated with a high proportion of metastatic disease at the time of diagnosis, contributing to very low rates of pancreaticoduodenectomy resection, and therefore low rates of tissue diagnosis.

Despite its aggressive nature, there remains a gap in the understanding of sarcomatoid carcinoma of the common bile duct with only a few documented cases. There is a lack of robust data on its clinical presentation, optimal management strategies, and prognostic factors, highlighting the need for additional case reports and future studies.

The aim of this case report is to provide insight into the aggressive behavior of sarcomatoid carcinomas and to emphasize the critical importance of early diagnosis and timely surgical management. Our case specifically highlights the importance of the lack of elevated tumour markers such as CA19-9 and CEA which may initially lead clinicians away from considering malignancy. Additionally, it highlights the necessity of advancing molecular characterization of these rare tumours to improve diagnostic precision and inform the development of more effective, targeted therapeutic strategies.

## Case presentation

A 72-year-old Caucasian male was admitted to the general surgery ward as an emergency to a district general hospital, presenting with four weeks of painless obstructive jaundice. The patient has previously been healthy with no diagnosed health conditions, no routine medications, no relevant family history and had not previously undergone any previous operations.

**Fig. 1:**
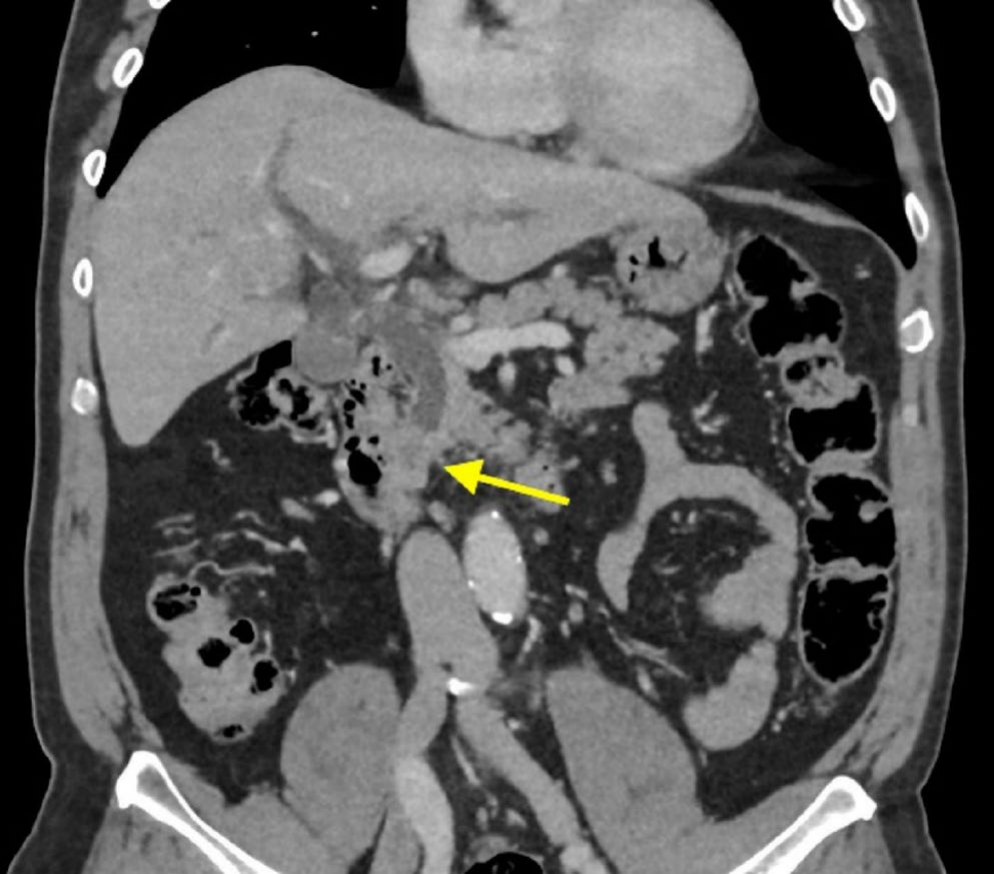
Coronal portal venous phase CT showing abrupt truncation of the distal CBD with 2.4cm stricture/intraluminal soft tissue which extends to the level of the ampulla, as indicated by the arrow. Associated moderate upstream intra and extrahepatic biliary tree dilatation is present

A thoraco-abdominopelvic computed tomography (CT) scan revealed a 15 mm filling defect within the distal common bile duct, accompanied by dilatation of both intrahepatic and extrahepatic biliary ducts (Fig. 1). There was no pancreatic duct dilation or evidence of a pancreatic mass. No other significant abnormalities were found on the CT scan.

**Fig. 2:**
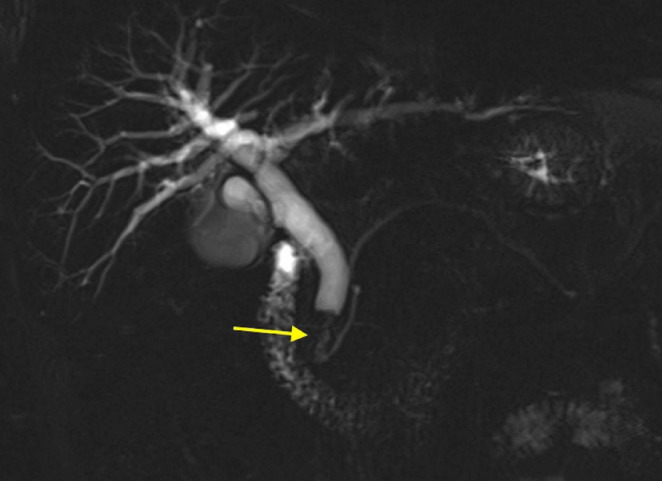
Coronal T2 SPACE MRI confirming distal CBD stricture as indicated by the arrow, with upstream biliary tree dilatation. Normal calibre main pancreatic duct suggests the mass originates within CBD rather than from the pancreas or ampulla

Magnetic resonance cholangiopancreatography (MRCP) confirmed biliary duct dilatation, revealing a solid mass measuring 19 mm longitudinally and 13 mm transversely, raising suspicion for malignancy (Fig. 2). An endoscopic retrograde cholangiopancreatography (ERCP) was attempted twice but was unsuccessful due to the inability to cannulate the common bile duct due to tumour involvement. Following this, the patient was transferred to our unit at Glasgow Royal Infirmary, after 10 days from initial admission at the district general hospital.

Laboratory tests on admission revealed significant abnormalities: alkaline phosphatase was elevated at 215 U/L, total bilirubin was markedly increased at 439 µmol/L, AST was 140 U/L, ALT was 164 U/L, and albumin was low at 27 g/L. Serum levels of carcinoembryonic antigen (CEA) and carbohydrate antigen (CA) 19-9 were within the reference range. The amylase level was mildly elevated at 157 U/L, and C-reactive protein (CRP) was raised at 39 mg/L. These results are presented in Table 1.

**Table 1:** Laboratory and tumour marker results obtained upon the patient’s admission to our unit, prior to stenting

Test	Result	Normal Range	Abnormality
Alkaline Phosphatase	215 U/L	40–150 U/L	Elevated
Total Bilirubin	439 µmol/L	< 21 µmol/L	Markedly Elevated
AST	140 U/L	8–33 U/L	Elevated
ALT	164 U/L	4–36 U/L	Elevated
Albumin	27 g/L	35–55 g/L	Low
Carcinoembryonic Antigen (CEA)	1.9 ug/L	0.0–5.0.0ug/L	Within Normal Range
Carbohydrate Antigen (CA) 19-9	16 kU/L	< 37 kU/L	Within Normal Range
Amylase	157 U/L	40–140 U/L	Mildly Elevated
C-Reactive Protein (CRP)	39 mg/L	< 10 mg/L	Elevated

Consequently, an endoscopic ultrasound-guided stenting of the bile duct was performed using a lumen opposing selfexpanding Metal Stent (SEMS), resulting in the complete resolution of bilirubin levels and a marked improvement in albumin levels to 33 g/L. We conclude that the improvement in serum albumin levels following stenting is attributable to the resolution of the temporary acute phase response caused by obstruction by the polypoid mass, which had previously contributed to the mildly low albumin levels. The patient clinically improved following stenting and was discharged from our unit 6 days after admission. He did not develop renal impairment, coagulopathy, or change in neurological status throughout his admission.

Magnetic Resonance Imaging (MRI) of the liver showed no evidence of metastasis. Given the high suspicion of malignancy and without tissue diagnosis, the patient underwent a classic Whipple resection (pancreaticoduodenectomy), 6 weeks post-discharge of his acute admission. During the operation, no metastasis was identified, but extensive inflammation of the portal structures and intrahepatic biliary tree stones were noted. The surgery had proceeded without the use of neoadjuvant chemotherapy or radiotherapy following multi-disciplinary team (MDT) decision, as the tumour was perceived resectable, and the patient was deemed fit and healthy with no comorbidities.

Pathological evaluation of the specimen demonstrated a Whipple pancreaticoduodenectomy specimen comprising stomach (40 mm along the greater curvature and 30 mm along the lesser curvature), small bowel (190 mm), pancreatic head (100 × 55 × 20 mm), attached common bile duct (40 mm), and cystic duct (25 mm). A separate gallbladder measuring 75 × 30 × 30 mm and omentum measuring 330 × 95 × 15 mm were also submitted.

On sectioning, a pale tumor was identified centered in the distal common bile duct, measuring 34 × 10 × 25 mm, with focal extension into the pancreas. A metallic lumen stent was present within the proximal common bile duct. The tumor exhibited a nodular configuration with solid architecture, demonstrating exophytic growth into the bile duct lumen and extension into the bile duct wall to a depth of 7.4 mm. Gross photographs of the resected specimen were unavailable due to institutional archiving limitations.

**Fig. 3:**
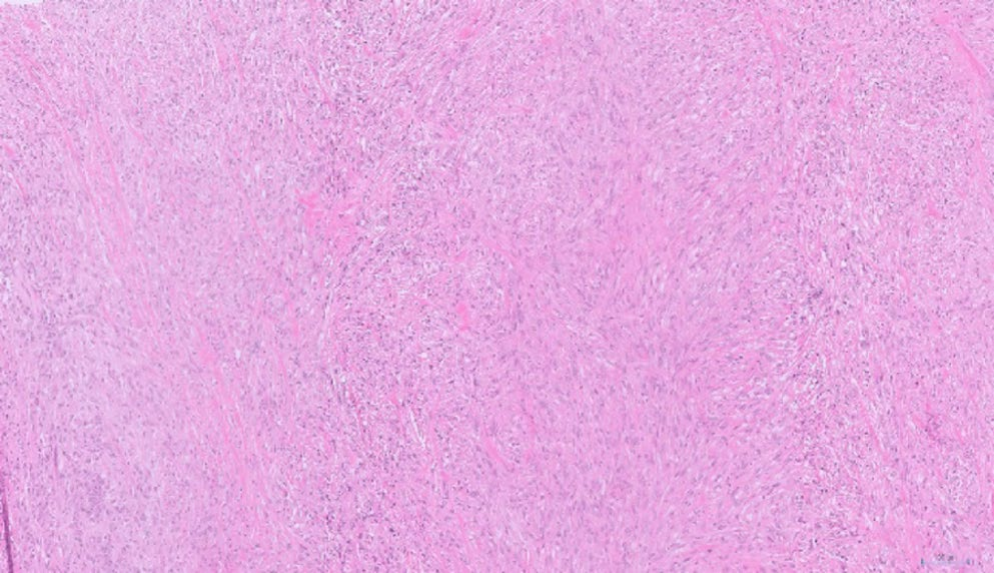
This micrograph demonstrates a tumour predominantly composed of spindle cells with pleomorphic hyperchromatic nuclei. The architecture indicates sarcomatoid differentiation, with regions showing ambiguous glandular structures that are challenging to distinguish from entrapped background epithelium

Histological examination revealed large, pleomorphic, spindle-shaped cells, along with angulated glands lined by biliary epithelium, exhibiting nuclear enlargement and prominent nucleoli (Fig. 3). Distinguishing between areas of glandular differentiation and entrapped background epithelium proved challenging. Specimen confirmed an R0 resection. A total of 18 regional lymph nodes were retrieved and histologically examined, including a separately submitted station 8 A lymph node. No metastatic carcinoma was identified in any of the examined nodes (0/18), corresponding to pN0 disease.

**Fig. 4:**
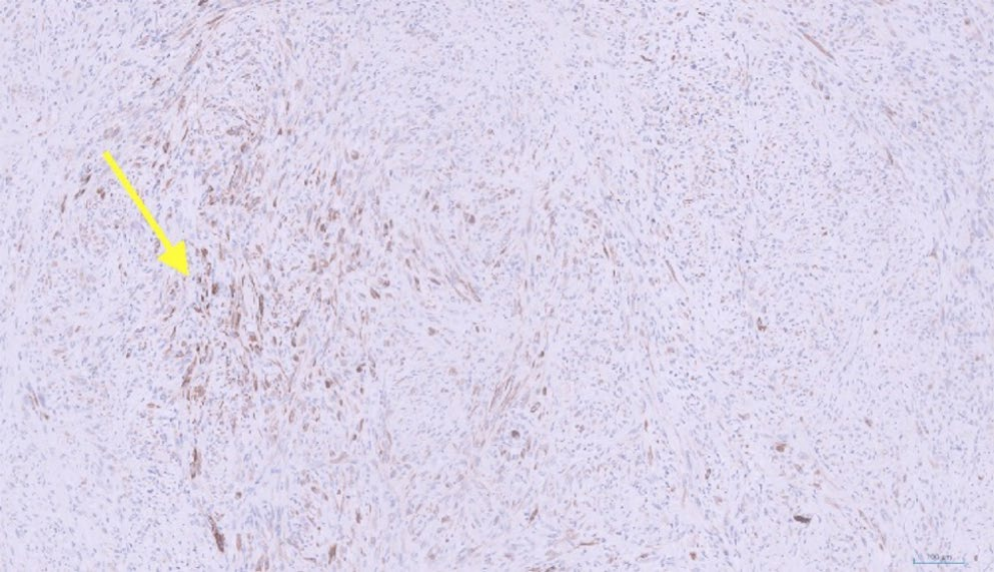
Immunohistochemical staining for AE1/3 demonstrates expression of the gene by spindle cells, confirming epithelial origin. The arrow points at a group of spindle cells that show strong expression of AE1/3

**Fig. 5:**
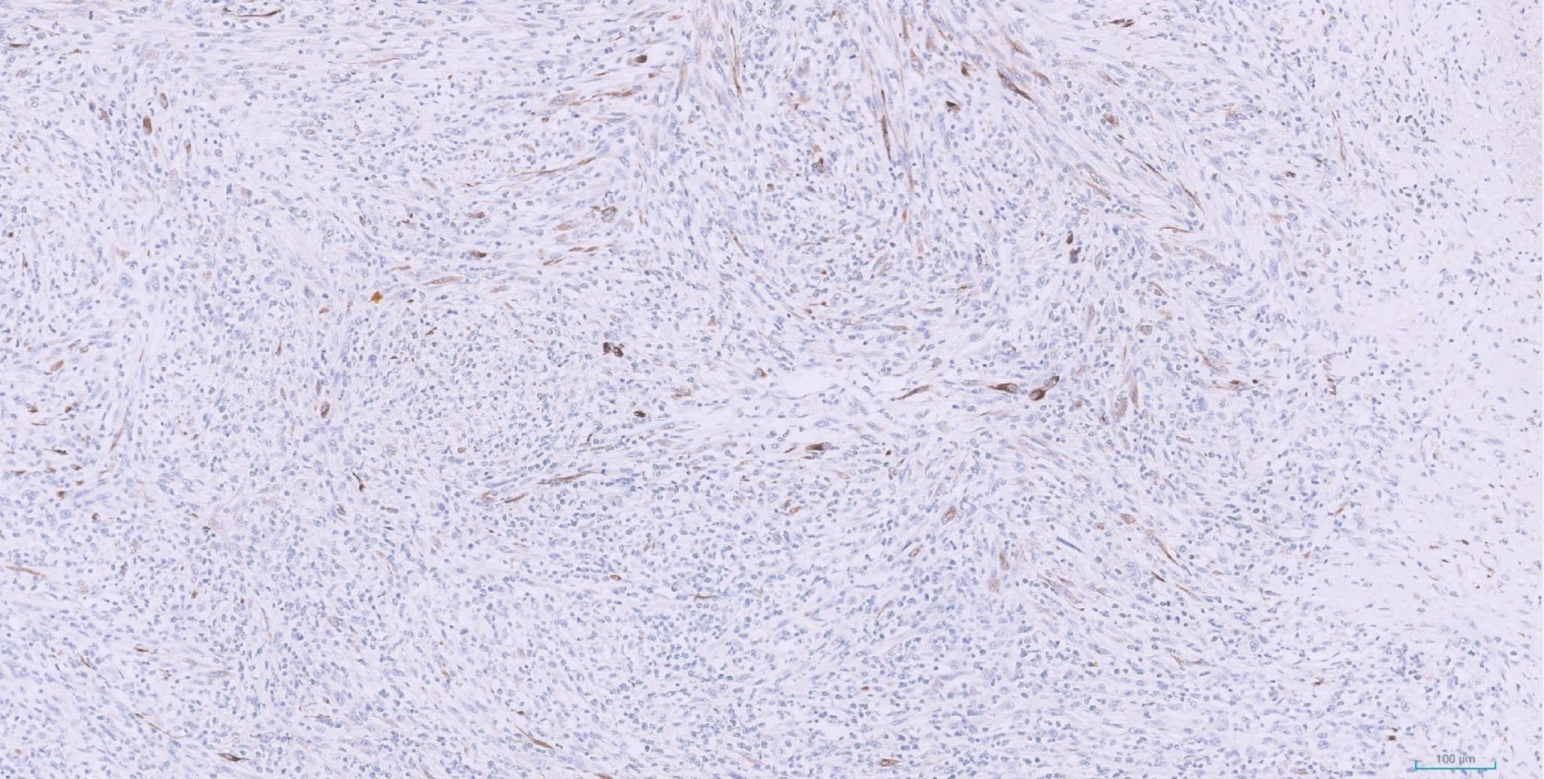
Immunohistochemical staining for CAM 5.2 demonstrates expression of the gene by spindle cells, confirming epithelial origin

Immunohistochemical staining showed patchy expression of AE1/3 (Fig. 4) and CAM5.2 (Fig. 5), with focal cytokeratin MNF16 positivity, while all other markers; including CD34, CD56, Desmin, DOG1, MyoD1, S100, smooth muscle actin (SMA), STAT6, synaptophysin, anaplastic lymphoma kinase (ALK), CD1a, CD68, and langerin were negative.

The extended immunohistochemical panel was employed to exclude important differential diagnoses. Negative ALK staining ruled out inflammatory myofibroblastic tumor [8], while STAT6 negativity excluded solitary fibrous tumor [9]. The absence of CD1a and langerin expression argued against histiocytic or dendritic cell neoplasms [10, 11], and negative lymphoid markers excluded lymphoma. Retained epithelial marker expression, including AE1/3 and CAM5.2, in combination with the histomorphological features, supported the diagnosis of sarcomatoid carcinoma. The lack of sarcomatous markers on staining directed us towards excluding carcinosarcoma as a diagnosis. The immunohistochemistry staining results are shown in Table 2.

**Table 2:** Immunohistochemical (IHC) staining profile of the resected specimen, summarising the expression of various markers, including cytokeratins, mesenchymal markers, neural markers, and other lineage-specific markers, detailed along with the corresponding antibody clone, manufacturer, heat-induced epitope retrieval (HIER) method, and dilution used

Marker (Kit/Company)	Expression	Antibody	Clone	Manufacturer	HIER	Dilution
AE1/3	Positive (Patchy)	NCL-L-AE1/AE3-601	AE1/AE3	LEICA	ER2(20)	1:200
CAM5.2	Positive (Patchy)	452 M-98	CAM5.2	CELL MARQUE	ER1(20)	RTU
Cytokeratin MNF16	Positive (Focal)	M0821	MNF-116	DAKO	ER2(20)	1:400
CD34	Negative	NCL-L-END	QBEN10	LEICA	ER1(20)	1:50
CD56	Negative	NCL-L-CD56-504	504	LEICA	ER2(20)	1:75
Desmin	Negative	NCL-L-DES-DER11	DE-R-11	LEICA	E1(10)	1:500
DOG1	Negative	NCL-L-DOG-1	K9	LEICA	ER2(20)	1:100
MyoD1	Negative	M3512	5.8 A	DAKO	ER2(20)	1:25
S100	Negative	NCL-L-S100-167	EP32	LEICA	ER2(20)	1:800
SMA	Negative	PA0943	ASM-1	LEICA	ER2(5)	RTU
STAT6	Negative	SC-374,021	D-1	SANTA CRUZ	ER2(20)	1:800
Synaptophysin	Negative	M7315	DAK-SYNAP	DAKO	ER1(20)	1:100
ALK	Negative	NCL-L-ALK	5A4	LEICA	ER1(20)	1:100
CD1a	Negative	NCL-L-CD1A-235	MTB1	LEICA	ER2(20)	1:40
CD68	Negative	M0876	PGM1	DAKO	ER2(20)	1:200
Langerin	Negative	NCL-L-LANGERIN	POLY	LEICA	ER2(20)	1:200

**Key-** ALK: anaplastic lymphoma kinase; DOG: discovered on gastrointestinal stromal tumour; SMA: Smooth Muscle Actin

The patient was diagnosed with Grade 3 sarcomatoid carcinoma, reflecting poor histologic differentiation, and stage T2 according to the TNM staging system of the American Joint Committee on Cancer (AJCC)/Union for International Cancer Control (UICC), 8th edition, for extrahepatic cholangiocarcinoma [12]. Chronic cholecystitis was observed in the gallbladder, along with a focal pancreatic intraepithelial neoplasia in the background pancreas.

The patient experienced no significant postoperative complications and was discharged 18 days post-surgery. As of the 16-month follow-up, there has been no recurrence of the disease, with the patient having returned to normal activities.

## Discussion

For our article, we performed a targeted literature review. To refine our approach, we utilized key references from two review papers: Lee et al. (2016) [13] and incorporated more recent articles from Yao et al. (2023) [14]. Although these articles were describing cases of carcinosarcoma, they included cases of sarcomatoid carcinoma in their reviews; we suspect due to their clinical and prognostic similarity. From these articles, we excluded fourteen specific papers: two were not available in English, one focused on sarcomatoid carcinoma arising from the intrahepatic bile duct, one was a case of sarcomatoid carcinoma of the intrapancreatic duct, and ten were carcinosarcomas. The previous eight cases of common bile duct sarcomatoid carcinoma we identified are presented in Table 3.

**Table 3:** Past cases of sarcomatoid carcinoma of the common bile duct described in the literature, including patient details, tumour characteristics, treatments, and outcomes

Author (year) (country)	Gender/age	Presentation	Investigation/tumour markers	Gross/size	Surgery	(C)RT	Pathology	IHC- stain	Previous HPB disease/intervention	Prognosis
Yoon et al. (2004) (Korea) [15]	Male, 78	Abdominal pain and obstructive jaundice	CT, PTC	Infiltrative, 40 mm	PD	Not used	Biphasic pattern of carcinomatous and sarcomatous components merging into another	**Positive**: CEA; Cytokeratin; NSE; PCNA; p53; SMA; Vimentin **Negative**: Bcl-2; Chromogrnin; Desmin; HMB45; Ki-67; Neurofilament; Synaptophysin; S100	None reported	Died at 5 days post-operatively due to cardiac complications
Jang et al. (2005) (Korea) [16]	Female, 68	Abdominal pain, anorexia, and weight loss	CT, EUS. Raised CA19-9. CEA and AFP within reference range	Polypoidal, 35 mm	PD	Not used	Biphasic pattern of predominantly sarcomatous and focally carcinomatous components	**Positive**: CEA; Cytokeratin; Vimentin **Negative**: C-kit; Desmin; Myoglobin; S100; SMA	None reported	Alive and “doing well” at 1 year post-operatively
Zhang et al. (2017) (China) [17]	Female, 51	Abdominal pain, obstructive jaundice, and weight loss	CT, EUS	Infiltrative, 38 mm	PD	3 cycles of Cisplatin and Gemcitabine chemotherapy	Biphasic pattern of carcinomatous and sarcomatous components and necrotic regions	**Positive**: AE1/AE3; CEA; CK18; CK19; CK7; Vimentin **Negative**: Desmin; Myoglobin; SMA; S100	Chronic hepatitis B	3 years disease-free
Kim et al. (2019) (Korea) [18]	Female, 65	Jaundice and abdominal pain	CT, ERCP, raised CA19-9. CEA and AFP within reference range	Infiltrative, 35 mm	PD	Not used	Biphasic pattern of predominantly sarcomatous and focally carcinomatous components	**Positive**: Pan-cytokeratin; vimentin **Negative**: Desmin; SMA	None reported	Died at 23 days post-operatively due to hepatic failure secondary to metastasis
Chan et al. (2023) (Canada) [19]	Female, 70s (unspecified)	Painless jaundice, decreased appetite, and fatigue	CT, MRCP/ERCP	Na, 47 mm	PD	Not used	Biphasic pattern of carcinomatous and sarcomatous components as well as squamous differentiation and sarcomatoid malignant peripheral nerve sheath tumour like areas	**Positive**: AE1/AE3; Calretinin; CD34; CD117; CK5; CK8/18; EMA; p63; synaptophysin; Vimentin **Negative**: ALK5A4; CDX2; CK7; CK20; CK19; Claudin; DOG; H3K27me3; MDM2; SOX10; S100; WT1	Open cholecystectomy	Died at 14 weeks post-operatively from perforated viscus, gastroduodenal leak and diffuse bowel ischaemia
Marques et al. (2023) (Portugal) [20]	Male, 79	Abdominal discomfort and obstructive jaundice	CT, MRCP, raised CA 19-9, CEA within reference range	Infiltrative, 12 mm	PD	Not used	Biphasic pattern of diffuse sheets of poorly differentiated carcinomatoid and sarcomatous components	**Positive**: CK7	None reported	2 years disease free
Nagata et al. (2024) (Japan) [21]	Male, 72	Obstructive jaundice	EUS, CT, MRI, ERCP, CEA and CA19-9 within reference range	Infiltrative, 32 mm	PD	Not used	Biphasic patten of sarcomatous and carcinomatous components, with a focus of adenocarcinoma near the CBD lumen. Slight infiltration into the pancreatic parenchyma, ki-67 index exceeded 80%	**Positive**: AE1/AE3; CAM 5.2; CK19; Vimentin **Negative**: CD34; Desmin; Myoglobin; S100; SMA	None reported	Intra-abdominal lymph node recurrence at 2 months, hepatic metastasis at 3 months; died at 7 months
Yao et al. (2024) (China) [22]	Male, 70	Obstructive jaundice	CT, raised CA19-9, raised CEA	Na, 10 mm	PD	Carelizumab immunotherapy with 6 cycles of Gemcitabine and Cisplatin	Biphasic pattern with predominant moderately differentiated adenocarcinoma component and some sarcomatous regions	**Positive**: CKpan; SMARCA4; Vimentin **Negative**: MyoD1 antibody; Myogenin; SMARCB1	None reported	Alive at 6 months post-operatively

**Key**- AFP: Alpha-fetoprotein; CDX: caudal type homeobox; DOG: discovered on gastrointestinal stromal tumor EMA: Epithelial Membrane Antigen; EUS: Endoscopic Ultrasound; MDM: mouse double minute; NSE: Neuron-Specific Enolase; PCNA: Proliferating cell nuclear antigen; PD: Pancreaticoduodenectomy; PTC: Percutaneous Transhepatic Cholangiography; SMA: Smooth Muscle Actin; SOX: SRY-related HMG-box; WT1: Wilms tumour gene

To ensure adequate literature saturation, we performed a PubMed search using the term “sarcomatoid carcinoma bile duct”; identifying Nagata et al. (2024) [21] and Yao et al. (2024) [22] as the only relevant recent publications, as included in our table. The remaining results predominantly covered pancreatic and gallbladder cases.

Carcinosarcomas were excluded from our review because they are pathologically distinct from sarcomatoid carcinoma. Sarcomatoid carcinoma is a malignant epithelial tumor exhibiting sarcomatoid (spindle cell) features while retaining epithelial marker expression (e.g., AE1/3, CAM5.2, MNF16). In contrast, carcinosarcoma is a biphasic malignant neoplasm composed of both epithelial (carcinomatous) and mesenchymal (sarcomatous) components. As such, sarcomatoid carcinoma of the bile ducts is a rare subtype of cholangiocarcinoma that exhibits mesenchymal molecular and histopathological features.

Incorporating our case into the existing literature, sarcomatoid carcinoma of the common bile duct appears to have an even distribution between genders, with no clear sex predilection; as 55.6% of reported cases have occurred in males. All described cases involve adults, with the youngest patient reported at 51 years and the oldest at 79 years. Notably, 88.9% of cases have been observed in patients over the age of 65. Additionally, 66.7% of cases originated from Asia. We suggest that this is more indicative of variations in surgical and academic practices rather than a true difference in prevalence [23]. The extremely limited number of reported cases underscores the rarity of this entity and highlights the difficulty in establishing definitive epidemiologic patterns.

Additionally, all the cases, including ours, tested positive for epithelial markers AE1/3 and vimentin when stained, highlighting the value of using these stains for tumours that exhibit sarcomatoid features in histology. Moreover, pancreatoduodenectomy was the primary treatment approach in all cases, with only 22.2% incorporating adjuvant chemotherapy. As such, the MDT, taking the patient’s wishes into account, felt that the evidence base for adjuvant chemotherapy was lacking.

Based on the relevant literature, historically, the primary treatment approach for this condition has been surgical resection, either with or without (neo)adjuvant chemoradiotherapy. Reported disease-free survival (DFS) times have shown significant variability, ranging from as short as 23 days post-operatively [18] to over 3 years, with no follow-up beyond that point [17].

Evidence from gallbladder sarcomatoid carcinoma may provide insight into its potential relevance for common bile duct pathology. A study conducted at Sichuan University reviewed five cases of advanced gallbladder sarcomatoid carcinoma (stage III or higher) treated over a 20-year period [24]. Among these patients, the longest survival was observed in the one who underwent adjuvant chemotherapy after surgery, with an overall survival of 15 months and 12 months without disease progression [24]. In contrast, the remaining patients, who did not receive chemotherapy, had a maximum survival of only 2.6 months [24]. This highlights the importance of reporting additional cases of this exceedingly rare malignancy with the aim of better characterising patterns of (neo)adjuvant therapy use and associated outcomes.

In addition to contributing to the limited literature on sarcomatoid carcinoma of the common bile duct, our case also illustrates the limitations of ERCP in achieving a tissue diagnosis of malignancy. Our case is a common example of failure to achieve tissue diagnosis following ERCP due to the narrowing of the bile duct, which accounts for the very low sensitivity of both cytological brush (45%) and forceps biopsy (67%) [25, 26]. In such situations, endoscopic ultrasound-guided biliary drainage has emerged as a minimally invasive alternative with high technical and clinical success rates, and was therefore selected in this case [27].

Lastly, we acknowledge that the extreme rarity of sarcomatoid carcinoma of the common bile duct and the narrative nature of our review represent significant limitations of this study, restricting the ability to draw robust conclusions regarding prognosis and optimal management. Further accumulation of cases and long-term follow-up are required to better understand the behavior and outcomes of this tumor type. 

## Conclusion

Sarcomatoid carcinoma of the common bile duct is an exceptionally rare and aggressive malignancy that poses significant diagnostic and therapeutic challenges. Accurate diagnosis depends on careful histopathological assessment supported by immunohistochemical confirmation of epithelial differentiation. Surgical resection remains the cornerstone of management for localized disease and offers the best opportunity for disease control. However, due to the extreme rarity of this entity, prognostic factors and the role of (neo)adjuvant therapy remain poorly defined. The limited number of reported cases demonstrates considerable variability in clinical outcomes, underscoring the need for continued case reporting, longer follow-up, and collaborative efforts to better characterize disease behavior and guide future management strategies.

## Supplementary Information

Below is the link to the electronic supplementary material.

Supplementary Material 1

Supplementary Material 2

## Data Availability

All data generated or analysed during this study are included in this published article (and its supplementary information files).
