# Supplementary material for: Sarcomatoid carcinoma of the common bile duct: A case report and review of the literature
Source: Int Cancer Conf J. 2026 Mar 10;15(2):258–66. doi: 10.1007/s13691-026-00852-8 (PMC13038722; doi:10.1007/s13691-026-00852-8)
Supplement: Supplementary file 2 — Supplementary Material 2 [file 13691_2026_852_MOESM2_ESM.docx]

**Search Strategy on PubMed**

“Sarcomatoid Carcinoma Bile Duct”
